# Dynamics of the Water Circulations in the Southern South China Sea and Its Seasonal Transports

**DOI:** 10.1371/journal.pone.0158415

**Published:** 2016-07-13

**Authors:** Farshid Daryabor, See Hai Ooi, Azizan Abu Samah, Abolghasem Akbari

**Affiliations:** 1 National Antarctic Research Center, Institute of Postgraduate Studies, University of Malaya, 50603, Kuala Lumpur, Malaysia; 2 Institute of Ocean and Earth Sciences, Institute of Postgraduate Studies, University of Malaya, 50603, Kuala Lumpur, Malaysia; 3 Faculty of Civil Engineering and Earth Resources, University Malaysia Pahang, Lebuhraya Tun Razak, 26300 Gambang, Kuantan, Pahang, Malaysia; University of Vigo, SPAIN

## Abstract

A three-dimensional Regional Ocean Modeling System is used to study the seasonal water circulations and transports of the Southern South China Sea. The simulated seasonal water circulations and estimated transports show consistency with observations, e.g., satellite altimeter data set and re-analysis data of the Simple Ocean Data Assimilation. It is found that the seasonal water circulations are mainly driven by the monsoonal wind stress and influenced by the water outflow/inflow and associated currents of the entire South China Sea. The intrusion of the strong current along the East Coast of Peninsular Malaysia and the eddies at different depths in all seasons are due to the conservation of the potential vorticity as the depth increases. Results show that the water circulation patterns in the northern part of the East Coast of Peninsular Malaysia are generally dominated by the geostrophic currents while those in the southern areas are due solely to the wind stress because of negligible Coriolis force there. This study clearly shows that individual surface freshwater flux (evaporation minus precipitation) controls the sea salinity balance in the Southern South China Sea thermohaline circulations. Analysis of climatological data from a high resolution Regional Ocean Modeling System reveals that the complex bathymetry is important not only for water exchange through the Southern South China Sea but also in regulating various transports across the main passages in the Southern South China Sea, namely the Sunda Shelf and the Strait of Malacca. Apart from the above, in comparision with the dynamics of the Sunda Shelf, the Strait of Malacca reflects an equally significant role in the annual transports into the Andaman Sea.

## Introduction

**Fig 1:**
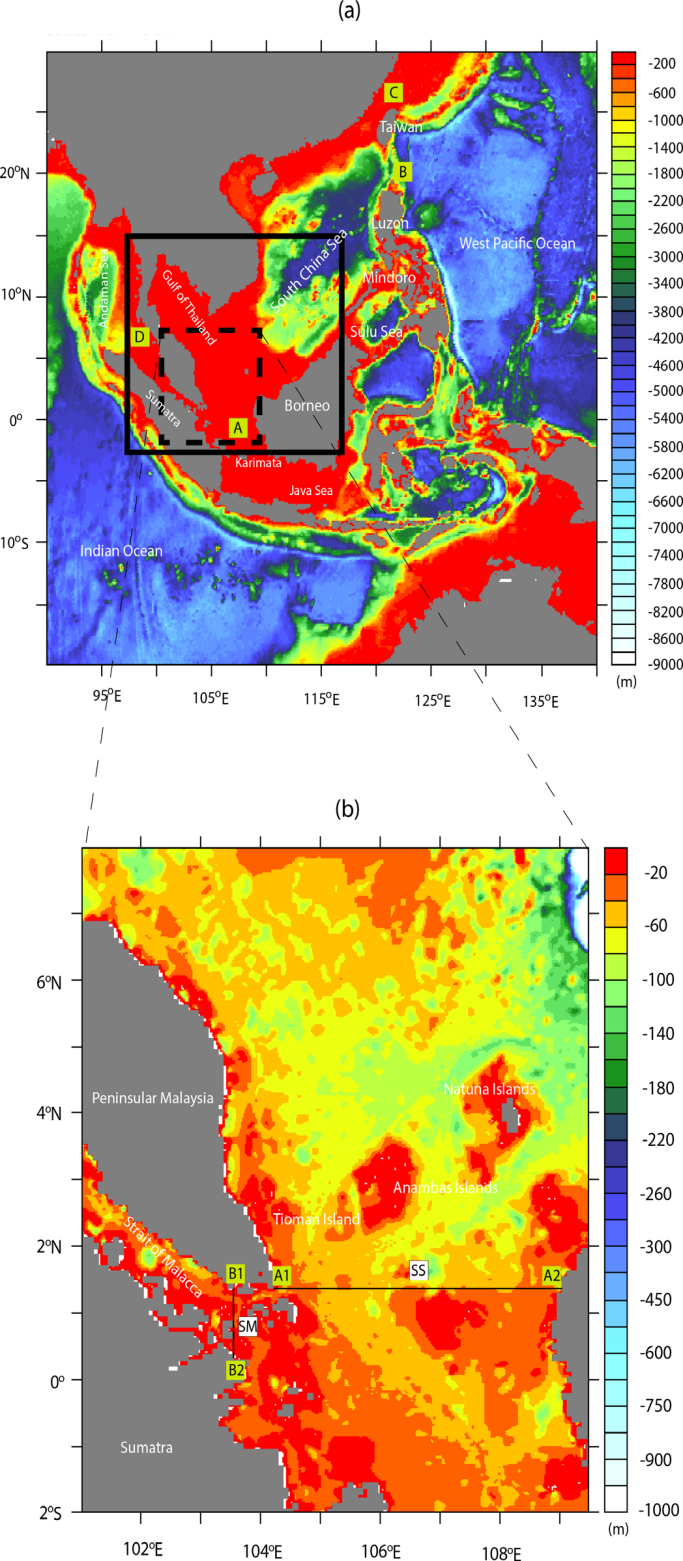
Bathymetry (in meters) for the outer domain and that for the inner domain (as black box) are shown in (*a*). The four lateral boundaries applied to simulation are represented by the letters A-D. The dashed box in (*a*) indicates the study area. Bathymetry of the study area with the black thin-line cross sections (A1A2 and B1B2) used for the transport budget analysis (transects SS and SM) shown in (*b*).

The South China Sea (hereafter abbreviated as SCS) is a marginal sea with a depth of about 4000–5000 m in the central region, becoming shallower towards the southern region with depths of 50–70 m in the Sunda Shelf. The Sunda Shelf (hereafter abbreviated as SS) here is fringed by Peninsular Malaysia, eastern Sumatra, Borneo Island, Java, and their surrounding smaller islands. The Strait of Malacca (hereafter abbreviated as SM) is located between the eastern Sumatra and the west coast of Peninsular Malaysia, linking the Southern South China Sea (hereafter abbreviated as SSCS) and the Andaman Sea. The minimum and maximum depths of the SM are in the southern and northern areas respectively (see Fig 1).

The complex bathymetry of the SSCS can affect significantly the dynamical processes of the region [1–4], especially in terms of water exchange through the SS and the SM and various transport pathways across the corresponding passages. As discussed by Daryabor *et al*. [1–2, 5] and noted by Liu *et al*. [4], the general water circulations in the SSCS are largely influenced by the monsoonal systems. It is characterised by oceanic cyclonic circulation in winter (December-February) and anticyclonic circulation in summer (June-August) with their respective embedded eddies of different horizontal scales.

During the southwest (summer) monsoon, bifurcation of the strong currents along the East Coast of Peninsular Malaysia (hereafter abbreviated as ECPM) towards the northeast of the SSCS and associated eddy formation affect not only the local flow fields but also the circulation patterns across the region. As the monsoon trough is located far north of the equatorial latitudes, the transport is thus caused by the anticyclonic shearing of the strong southeasterlies flowing mainly parallel to the ECPM, particularly in the presence of distant tropical storms or typhoons. However, during the northeast (winter) monsoon, as the monsoon trough is close to the equator in the SSCS, the transport mechanism is due to the cyclonic shear of the northeasterlies, especially strong in the presence of cold surges as a result of outburst of Siberian high from China.

Many studies have estimated transports in the SSCS from surface observations and numerical models as illustrated in Tables 1 and 2. Observational studies by Wyrtki, [6], Fang *et al*. [7], and Song, [8] show that there is volume outflow from the SSCS into the Java Sea during winter and inflow into the SSCS during summer. Model studies by Liu *et al*. [4], Cai *et al*. [9], Fang *et al*. [10–11], and He *et al*. [12], not only on volume transport (Table 1) but also salt and heat transports (Table 2), indicate large values of volume transport flowing into the Java Sea and the SSCS through the SS during winter and summer seasons respectively. Estimates of various transports as shown in Tables 1 and 2 vary widely, mainly due to the differences in model configurations and resolutions, especially when the shallow areas and pathway are connected to the channels and the adjacent seas [13].

**Table 1:** Estimates of the volume transport (10^6^ m^3^/s) through the SS and SM (bracketed) based on previous studies. Positive and negative values indicate outflow and inflow transports, respectively.

References	Approach	Method	HR^a^ (in km)	Winter	Summer	Annual
*Wyrtki*, [6]	Obs.	Ship Drift Data	-	+4.5	-3	-
*Fang et al*. [7]	Obs.	Acoustic Doppler Current Profiler (13 January to 12 February 2008)	-	+3.6	-	-
*Song*, [8]	Obs.	Satellite Sea Surface Height and Ocean Bottom Pressure Data	-	+7.5	-	-
*Cai et al*. [9]	Model	The LASG/IAP Climate Ocean Model (LICOM)	56×56	-	-	+2.26
*Fang et al*. [10]	Model	Modular Ocean Model (MOM) Version 2	333×333^b^ and 18×18^c^	-	-	+3.1[+0.5]
*Fang et al*. [11]	Model	Modular Ocean Model (MOM) Version 2	222×222^d^ and 18×18^e^	-	-	+1.16[+0.16]
*Liu et al*. [4]	Model	Bluelink ReANalysis (BRAN)	11×11(89)^f^	+4	-1	+1.42[+0.27]
*He et al*. [12]	Model	Bluelink ReANalysis Assimilated Data (BRAN)	10×10	+3.8	-0.9	+1.6

^a^ Horizontal Resolution

^b, d^ horizontal resolution for the outer domain (global)

^c, e^ for the inner domain (i.e., SCS and adjacent seas)

^f^ horizontal resolution along *X* axis, changing from 11 km south of 16.5°N and decreasing to 89 km near 24.6°N along *Y* axis for the SCS.

**Table 2:** Numerical estimations of the annual salt transport (10^9^ kg/s) and heat transport (10^15^ J/s) through the SS and SM based on previous studies.

References	Approach	Method	HR (in km)	SS		SM	
				ST^a^	HT^b^	ST	HT
*Fang et al*. [10]	Model	Modular Ocean Model (MOM) Version 2	333×333^c^ and 18×18^d^	+0.11	+0.35	+0.017	+0.06
*Fang et al*. [11]	Model	Modular Ocean Model (MOM) Version 2	222×222^e^ and 18×18^f^	+0.039	+0.11	+0.005	+0.02

^a, b^ salt and heat transports respectivrly

^c, e^ horizontal resolution for the outer domain (global)

^d, f^ for the inner domain (i.e., SCS and adjacent seas).

In view of the complexity of bathymetry and flow patterns in the SSCS, high-resolution regional ocean models are required for a better understanding of dynamical processes of the flow field to quantify the fluxes through the two principal passages (namely the SS and SM). Also, observational data in the SSCS are sparse as compared with those in the northern region of the SCS. This has limited the estimation of the various transports (such as freshwater, heat, and salt) in the SSCS. In particular, such estimations for the SM are virtually non-existent [4, 10–11]. Considering that the existing passages in the SSCS play an important role in regulating the transport exchanges between the Pacific and the Indian Ocean [4], it is necessary to have such estimations in spite of the limitations. On the other hand, in SSCS, computations of sea level anomaly and transport involve the use of barotropic and baroclinic velocities in the advection scheme of the model. Ezer *et al*. [14] show that, in their comparison between the Princeton Ocean Model (POM) and the Regional Ocean Modeling System (hereafter abbreviated as ROMS), most of the model differences in the solutions of oceanic processes are the result of the advection schemes due to pressure gradient errors which can cause changes of the order of a few tenths of centimeter in the sea level anomaly and 1×10^6^ m^3^/s in transport. Such pressure gradient error is reduced in ROMS by using the high order accurate pressure gradient scheme (i.e., Density Jacobian scheme with monotonized cubic polynomial fits), leading to the smallest error obtained for the barotropic and baroclinic velocities and thus the preferred use of the model in the SSCS.

Based on the above premise, the present study focuses on the understanding of the seasonal characteristics of the water circulations and associated dynamics of the various transports in connection with volume, freshwater, heat, and salt in the SSCS. This necessitates the use of the three dimensional Regional Ocean Modeling System with finer horizontal and vertical resolutions to comprehend the issues mentioned above. ROMS [15–16], being a split-explicit, free-surface ocean model, solves incompressible primitive equations using the Boussinesq and hydrostatic approximations [15–16]. To reduce the dispersion errors, a third-order upstream-biased, dissipative advection scheme is employed and this scheme also enhances the grid resolution accuracy [17]. As mixing is known to be attributed to the implementation of higher-order diffusive advection schemes [18], splitting of advection and diffusion can thus resolve spurious diapycnal mixing in the sigma-coordinates. The method used in the nested ROMS simulation is designed to retain the low dispersion and diffusivity capabilities of the original scheme.

In this paper, after the description of the model setup and data in Section 2, the dynamics of water circulations is discussed in Section 3. The seasonal variations of temperature and salinity are investigated in Section 4. Section 5 details the modelled transports in the SSCS and Section 6 concludes with the summary.

## Model Description and Data Sources

The ROMS developed in the institut de recherche pour le développement (http://www.romsagrif.org/index.php/) is used for simulating seasonal water circulations and transports. In this model, the outer domain covers from 20°S—30°N, 90°E—140°E with 50 km horizontal resolution while the inner domain with 9 km horizontal resolution stretches from 2.7°S—15°N, 97.2°E—116.7°E (Fig 1A).

For the present simulation, the ETOPO2 (see http://www.ngdc.noaa.gov), which is derived from depth soundings and satellite gravity observations [19] with a horizontal spatial bathymetry resolution of approximately 3 km, is used for both the outer and inner model domains. 30 vertical levels following the bathymetry but with a minimum depth (hmin) setting of 5 m at the shore are applied in both the domains. In connection with this, it is to be noted that during simulation the vertical mixing needs to be computed based on the non-local K-Profile Parameterization (KPP) scheme on the basis of the boundary layer formulation proposed by Large *et al*. [20]. The four open boundaries (defined by the letters A-D, see Fig 1A) at the north, south, east and west of both domains are specified. The model is integrated for 10 years in total using climatological data set with 3 years spin-up time to achieve quasi-steady state [2]. From the inner domain, respective averages of 7 individual values of each of the 12 months derived from years 4 to 10 are used to analyse the dynamics of the water circulations as well as its seasonal/annual transports in the SSCS. Full details of this implemented model as well as explanations on various model options used are available in Daryabor *et al*. [2].

The analyzed climatological fields of *in-situ* temperature [21] and salinity [22] from the World Ocean Atlas 2005 (WOA05) (http://www.nodc.noaa.gov/OC5/WOA05/pr_woa05.html) are set as initial and boundary conditions. The oceanic surface forces in terms of climatological monthly mean wind stress, surface freshwater flux (Evaporation minus Precipitation; hereafter abbreviated as (E-P)) and heat fluxes (refer to http://iridl.ldeo.columbia.edu/SOURCES/.DASILVA/.SMD94/.climatology/) are obtained from the Comprehensive Ocean-Atmosphere Data Set (COADS) [23]. The water circulations from ROMS are validated against the monthly mean currents of Ocean Surface Current Analyses-Real time with a horizontal resolution of 37 km (OSCAR; http://www.oscar.noaa.gov/) for the period 2000 to 2006. Similarly, the simulated climatological Sea Surface Temperature (SST) is compared with that from the Group for High Resolution Sea Surface Temperature (GHRSST) with a horizontal resolution of 5.5 Km for the same period (refer to http://podaac.jpl.nasa.gov/dataset/NCDC-L4LRblend-GLOB-AVHRR_OI). This product uses optimal interpolation (OI) for data from the 4 km Advanced Very High Resolution Radiometer Pathfinder Version 5 extracted by Reynolds *et al*. [24], and *in-situ* ship and buoy observations. In addition, the simulated Sea Surface Salinity (SSS) is validated against that from the hydrographic climatological data “HydroBase” version 2 with approximately 100 km horizontal resolution (refer to http://www.whoi.edu/science/PO/hydrobase/php/index.php) while the simulated seasonal sea surface height anomaly (SSHA) and surface geostrophic currents are compared with the mean value for the period 2000–2006 from the Archiving Validation and Interpretation of Satellite Oceanographic (AVISO) (refer to http://aviso.altimetry.fr/) data set. Lastly, the re-analysis ocean data set derived from Simple Ocean Data Assimilation (SODA; http://apdrc.soest.hawaii.edu/datadoc/soda_2.1.6.php) [25–26] are compared with the various transports estimated from the model.

## Seasonal Wind Stress and Water Circulations

### Seasonal wind stress

**Fig 2:**
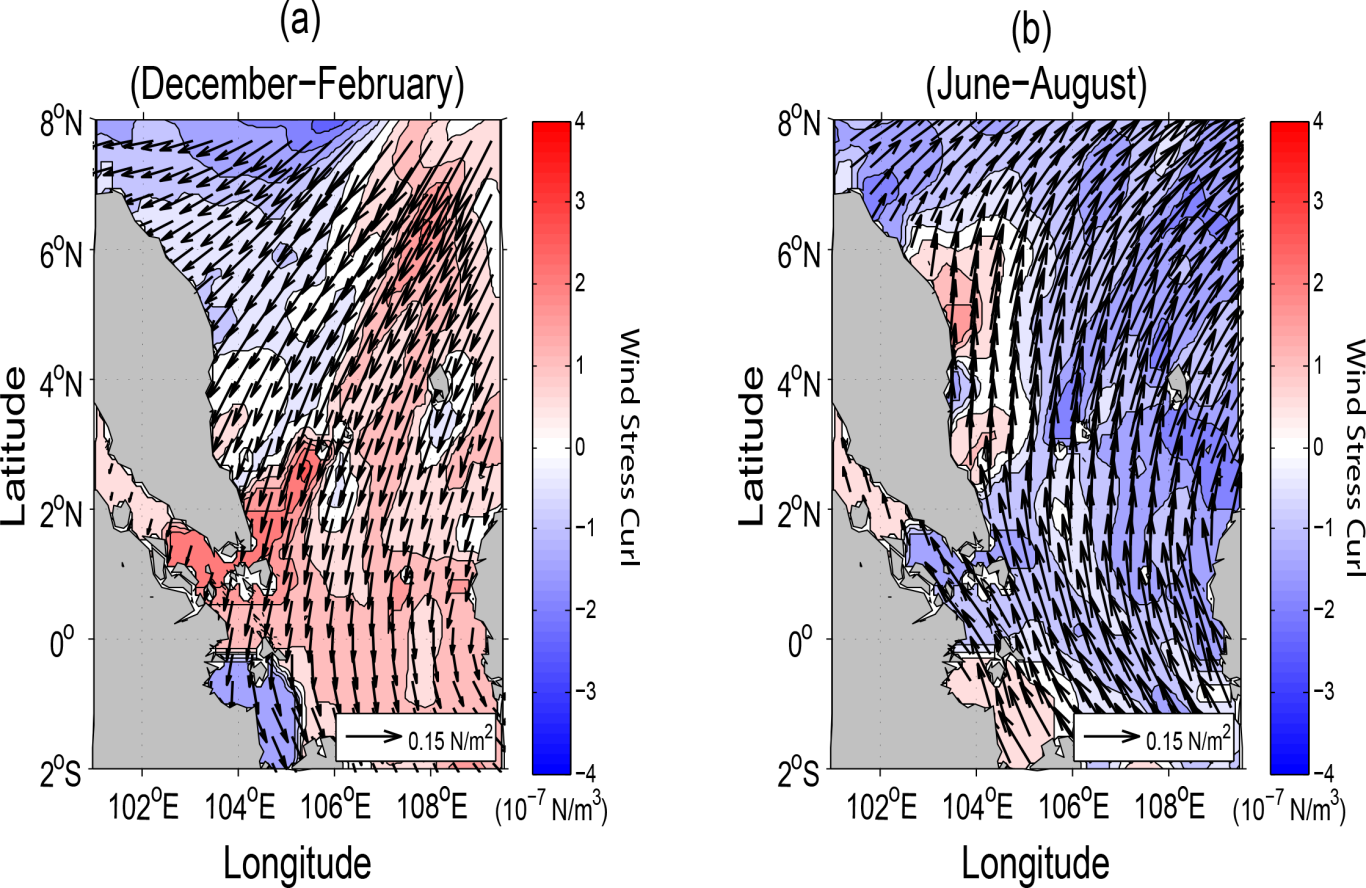
Vector of wind stress superimposed by corresponding wind stress curls (10^−7^ N/m^3^), (*a*) in winter and (*b*) summer, derived from the monthly climatological data set [23].

The seasonal wind stress fields and the corresponding wind stress curls in the SSCS are shown in Fig 2. During winter (December-February), wind stress is directed southwestwards over the entire domain and is reversed during the summer season (June-August).

The maximum magnitudes of the wind stress curl in the two monsoon seasons are approximately found across the central part of the domain. In particular, a negative wind stress curl is noted in the northwestern sector during winter and almost the entire domain during summer. Positive wind stress curl is seen in the southeastern sector of the domain during winter but only along the coastal region of the ECPM during summer. It is known that positive wind stress curl leads to the Ekman divergence during summer and the negative curl causes convergence during winter [2]. Obviously, the seasonal water circulations in the upper layers to be discussed in the subsequent section can be modified significantly by the seasonal wind stress patterns especially due to the small or negligible Coriolis force south of 5°N.

### Seasonal water circulations

**Fig 3:**
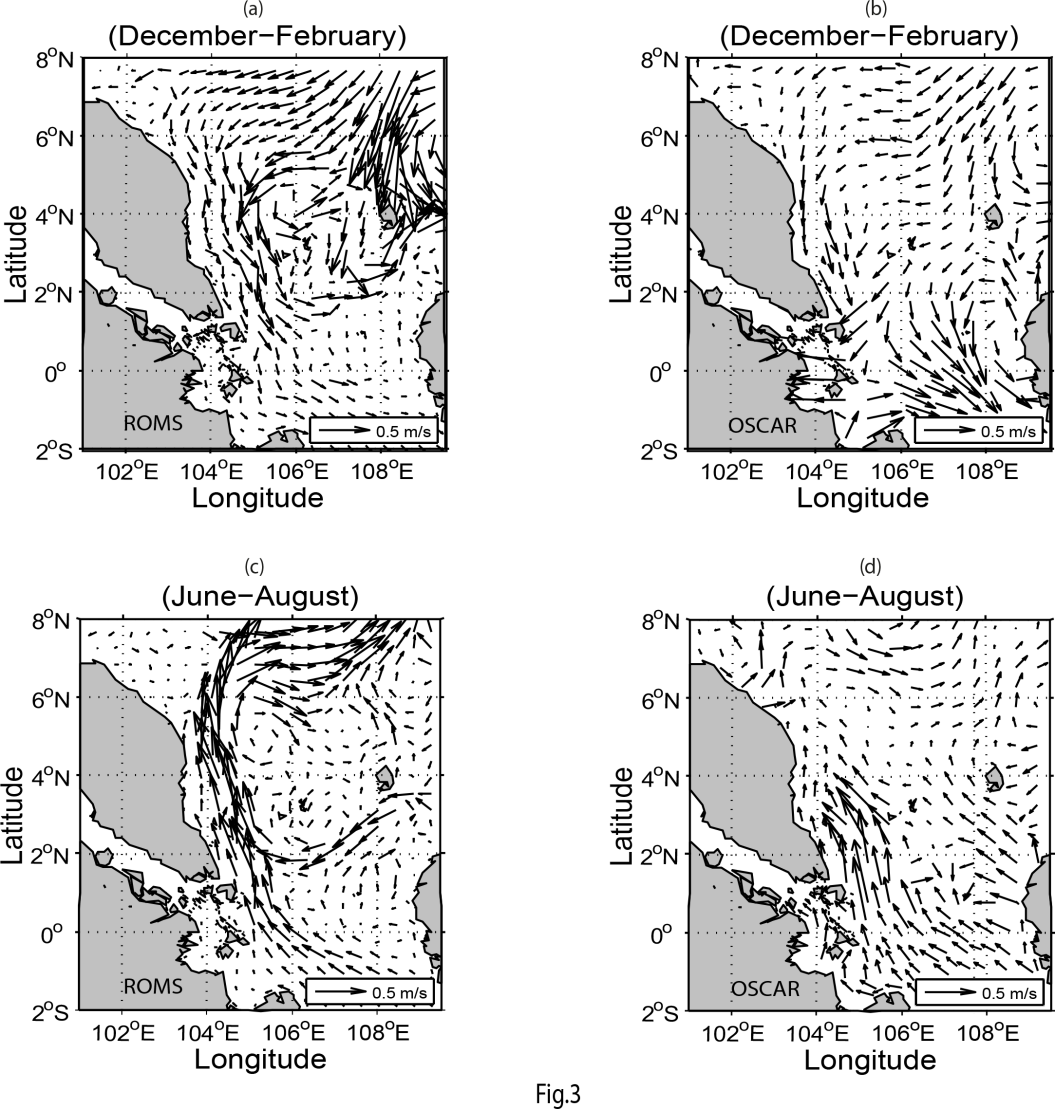
Seasonal surface water circulations: (*a*-*b*) for winter (December-February) and (*c-d*) for summer (June-August) derived from the ROMS and OSCAR respectively.

The mean seasonal surface currents of the simulated model and near-realtime global ocean surface currents derived from satellite altimeter and scatterometer data (OSCAR) for the winter (December-February) and summer (June-August) seasons are shown in Fig 3. As OSCAR data are near-realtime instead of climatology and its area coverage is global with coarse resolution as compared to that of the model, the simulated surface circulation patterns inclusive of the strong boundary currents along the ECPM are not exactly resembled those of OSCAR. As noted by Daryabor *et al*. [1], wind stress force can distinctly modify the magnitude of the currents. This is further enhanced by the resolution of the model and its interaction with the complex bathymetry in the region. In terms of eddy formation, it is mainly due to its interaction with the bathymetry [3]. Hence, this explains why significant difference in the current circulation patterns exists during both of the winter and summer seasons between ROMS and OSCAR due to not only the finer resolution of the ROMS model but also the unavailability of OSCAR data set close to the coast. Nevertheless, the cyclonic eddies during winter at north of the Natuna and Anambas Islands (see Island locations in Fig 1B) and the anticyclonic eddies during summer are the noticeable and distinct features in both the ROMS and OSCAR.

During summer, the western boundary current bifurcates from the ECPM at around 6°N (Fig 3C and 3D). This may be due to the formation of a pressure gradient and vorticity around the coastal region [2]. The water circulation patterns at depths of 30 to 50 m are similar to those near the surface [1, 27]. The cyclonic and anticyclonic eddies in the respective winter and summer seasons that exist north of the Natuna and Anambas Islands are due to the development of baroclinic instability in the corresponding areas effected by the dominant monsoonal winds [1–2, 28–29].

**Fig 4:**
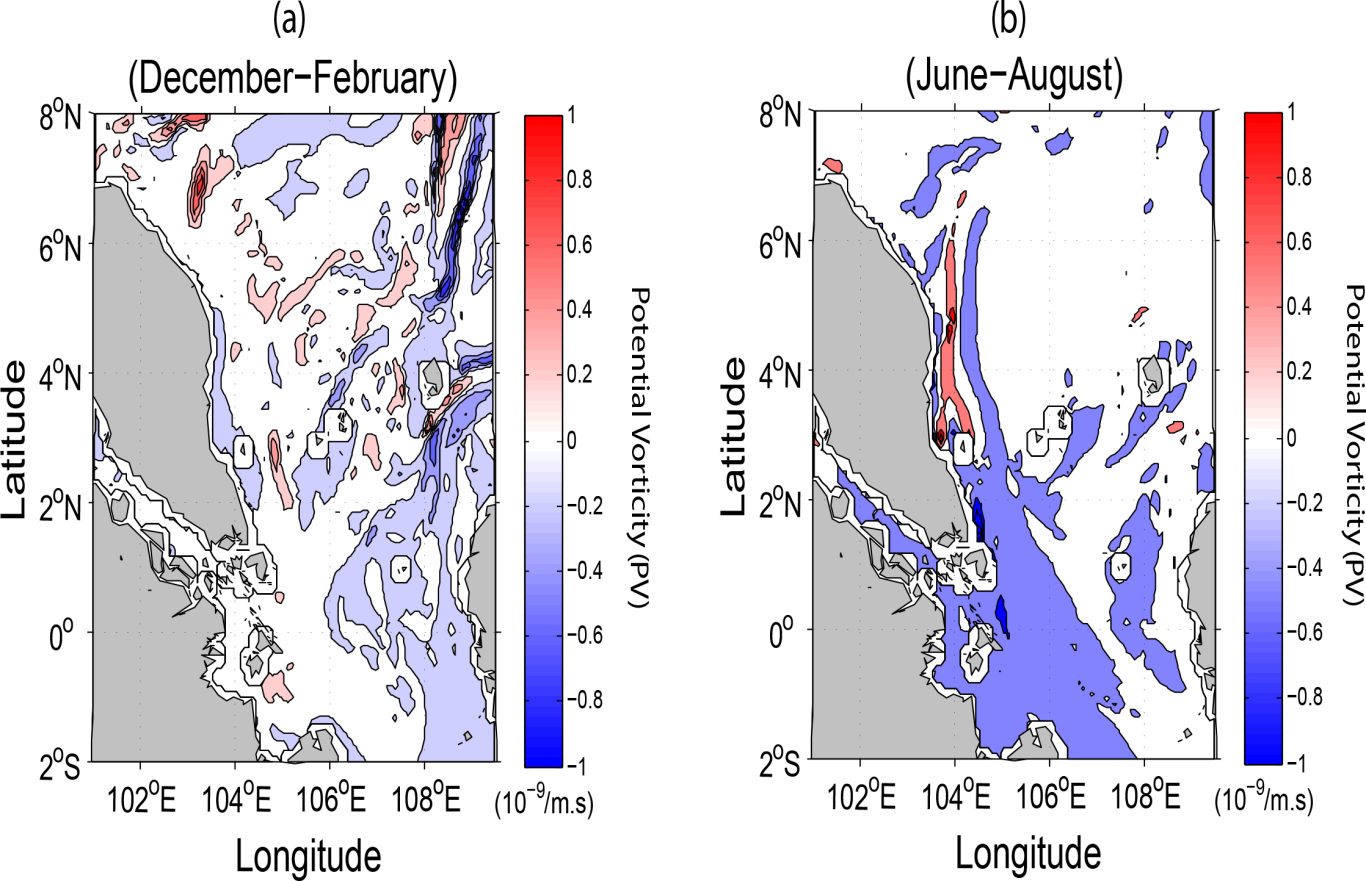
ROMS-derived seasonal surface potential vorticity (1/m.s) during (*a*) winter and (*b*) summer.

The persistent patterns with decreasing strength of the current flows and their embedded eddies at different depths can be explained by the conservation of potential vorticity as expressed by the Eq (1) below:

$$PV=\frac{\zeta +f}{h+\xi }$$ (1)

where *PV* denotes potential vorticity (1/m.s), *ζ* and *f* are relative and planetary vorticities (1/s) respectively, *h* and *ξ* (meter) are the respective total depth and sea surface height anomaly (hereafter abbreviated as SSHA) derived from the model. Assuming that *f* and *ξ* are constant at a specific location during a season, as *h* increases (with respect to the sea bottom), *ζ* must also decrease, leading to reduced strength of eddies so as to conserve potential vorticity. Similarly, the eastward veering of the coastal jet along the ECPM in the sea surface only during summer and not during winter can also be attributed to the conservation of *PV* as *f* increases northwards, leading to a large negative *PV* east of the jet core as shown in Fig 4.

By assuming the geostrophic balance, the geostrophic current is computed from the sea surface height anomaly (*ξ*) derived from the model using the following equation;

$$\left\{ \begin{array}{l} u_{g}=\frac{-g}{f}\frac{\partial \xi }{\partial y}\\ v_{g}=\frac{g}{f}\frac{\partial \xi }{\partial x} \end{array}\right.$$ (2)

where *u*_*g*_ and *v*_*g*_ in unit m/s are the zonal and meridional sea surface geostrophic components respectively, *g* (m/s^2^), the gravitational acceleration and *f* (1/s) is the Coriolis parameter.

**Fig 5:**
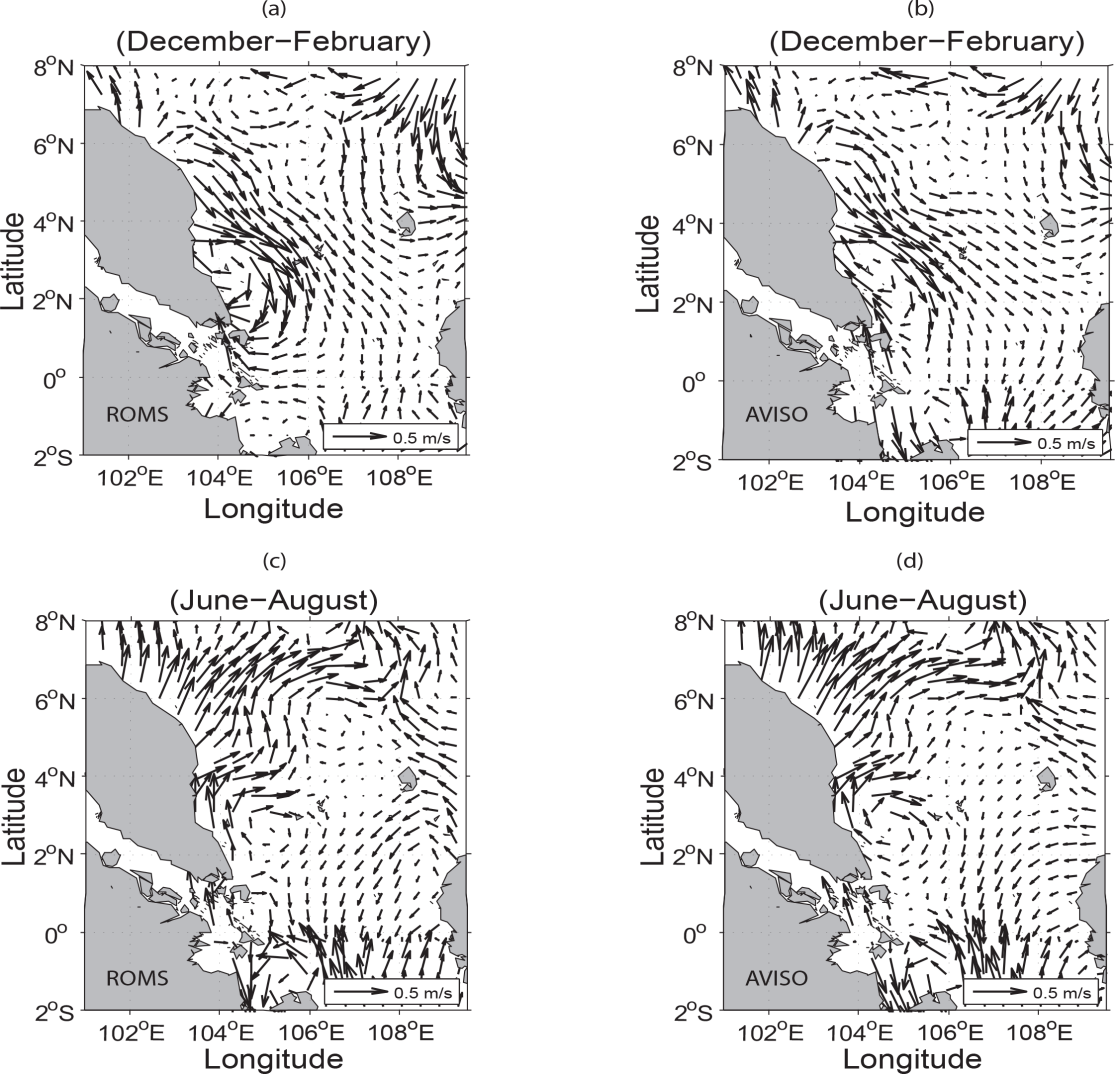
Seasonal surface geostrophic currents: (*a*-*b*) for winter and (*c*-*d*) for summer derived from the ROMS and AVISO respectively.

The seasonal geostrophic currents at the sea surface from both the model and AVISO for the winter and summer is shown in Fig 5. This figure shows that the circulation patterns, particularly the horizontal scales and the locations of eddies in the winter, are almost identical with the current patterns at sea surface as shown in Fig 3. Similarly, during summer, the surface geostrophic currents derived from the model and AVISO show clearly the bifurcation of coastal strong current at around 6°N. In particular, the anticyclonic eddies are also in accord with the sea surface currents as shown in Fig 3. Thus, it can be deduced that the bifurcation of the sea surface currents and the resultant eastward flow of water in summer are due mainly to the existence of dominant geostrophic currents in the region. In general, Figs 3 and 5 demonstrate that the water circulation patterns in the SSCS north of 5°N are dominated by the geostrophic currents because of the increasing Coriolis parameter (*f*) while those south of 5°N are solely controlled by the wind stress due to negligible *f*.

**Fig 6:**
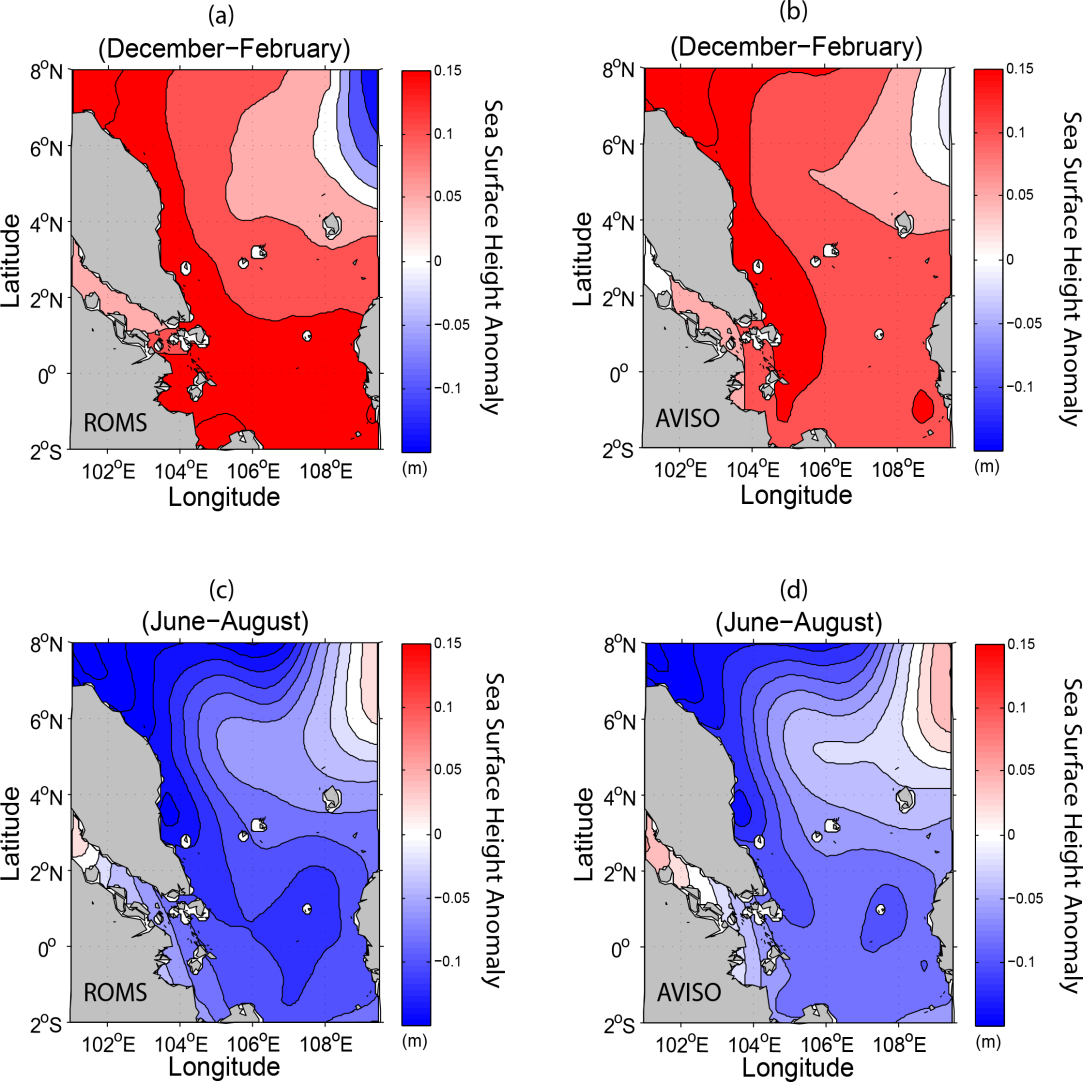
Same as Fig 5 but for seasonal sea surface height anomaly (m).

The sea surface height anomalies derived from the ROMS both in winter and summer are in good agreement with those obtained from the AVISO (Fig 6). The high and low SSHA patterns over the SSCS during both seasons are characterized by not only the respective predominant northeasterly and southwesterly flows on the water circulations in the upper layers but also their coincidence with the respective oceanic cyclonic and anticyclonic eddies in the northeastern domain. Apart from the increasing *f* north of 5°N that gives rise to geostrophic currents, the differences in SSHA that create the pressure gradient force are also the added factor for the resultant current flow patterns in the domain. This implies that the model is able to capture realistically the ocean dynamics in the SSCS.

## Seasonal Sea Surface Temperature and Salinity

**Fig 7:**
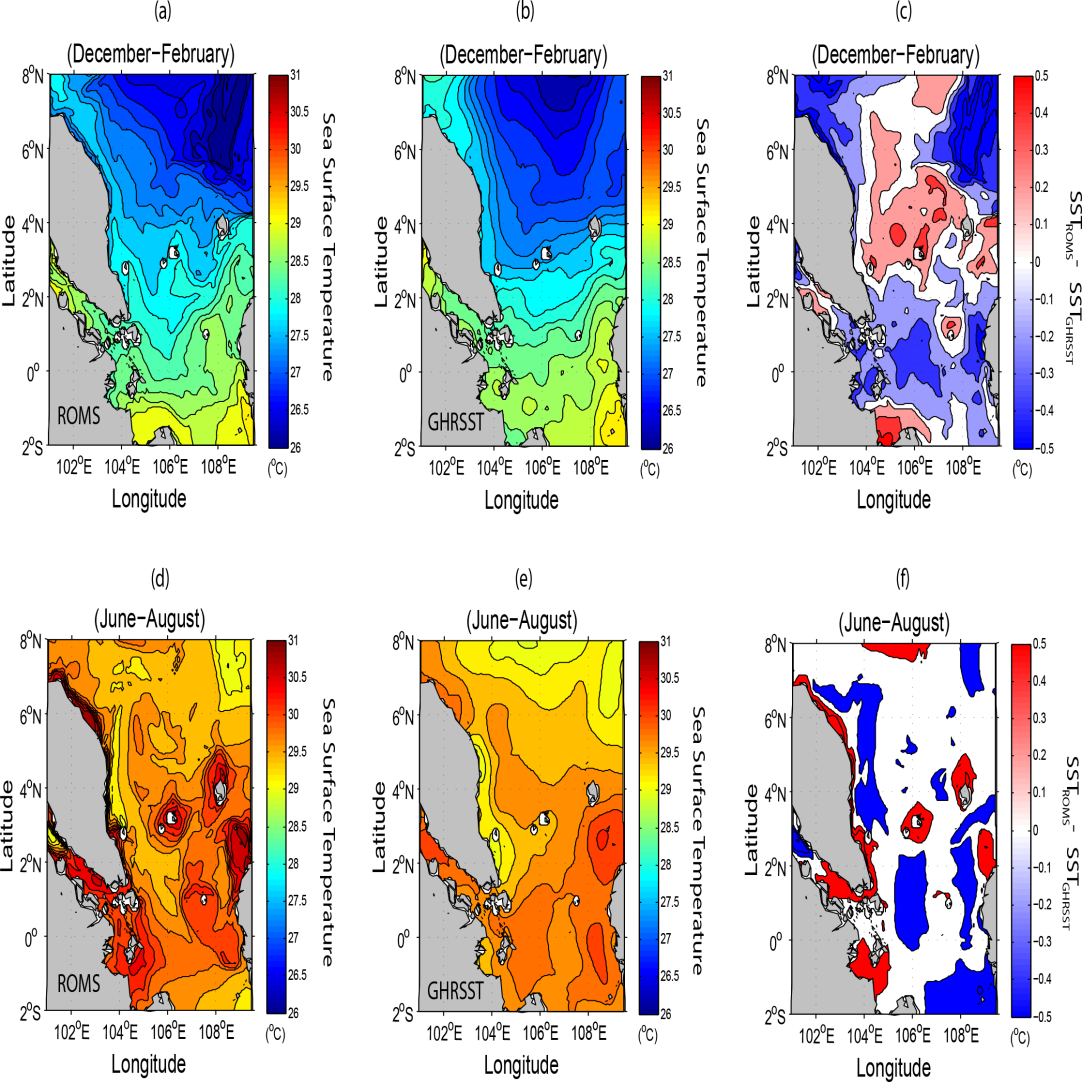
Seasonal variations in sea surface temperatures in °C; (*a*-*b*) for winter and (*d-e*) for summer derived from the ROMS and GHRSST respectively while (*c*) and (*f*) represent their respective differences.

The sea surface temperature (SST) variabilities during winter and summer are mainly controlled by the advecting current flowing towards or away from the SSCS [1–2, 30]. As shown in Fig 7, the model derived SST pattern is similar to that of the observed. The small difference in positive and negative values (as in (c) and (f) of Fig 7) as well as their distributions do indicate the existence of some differences between the modelled and observed patterns. These are due to (1) the finer horizontal and variable vertical resolutions of the configured model [2, 31], (2) the input of climatological instead of stronger near-real time wind data into the model especially when the latter interacts with the complex bathymetry particularly near the coastal areas, and (3) the result of modelled vertical turbulent diffusion times as well as the vertical advection or convection in the upper layer during the strong southwest monsoon. Nevertheless, from the model perspective, it is shown that advection of cold current from the northeast during winter leads to the relatively low SST (using 28°C as the threshold of equatorial SST) north of 1.5°N. This cold tongue extends from the southern China coast southwestwards towards the SSCS and along the ECPM. South of 1.5°N where the warmer SST persists is in fact the average position of the near equatorial trough and this warmer water is noted to flow outwards towards the Java Sea (Fig 7A and 7B). On the other hand, the SSTs in the SSCS during summer are generally 30°C on average (Fig 7D and 7E). This is due to the increased solar radiation and advection of warm waters from the southern region of the SSCS [1–2, 30, 32–33]. One significant feature that is reflected in both the model and observation is the existence of an elongated and narrow cold tongue along the ECPM with the temperature ranging approximately 28.5–29°C due to the occurrence of upwelling [1–2, 34].

**Fig 8:**
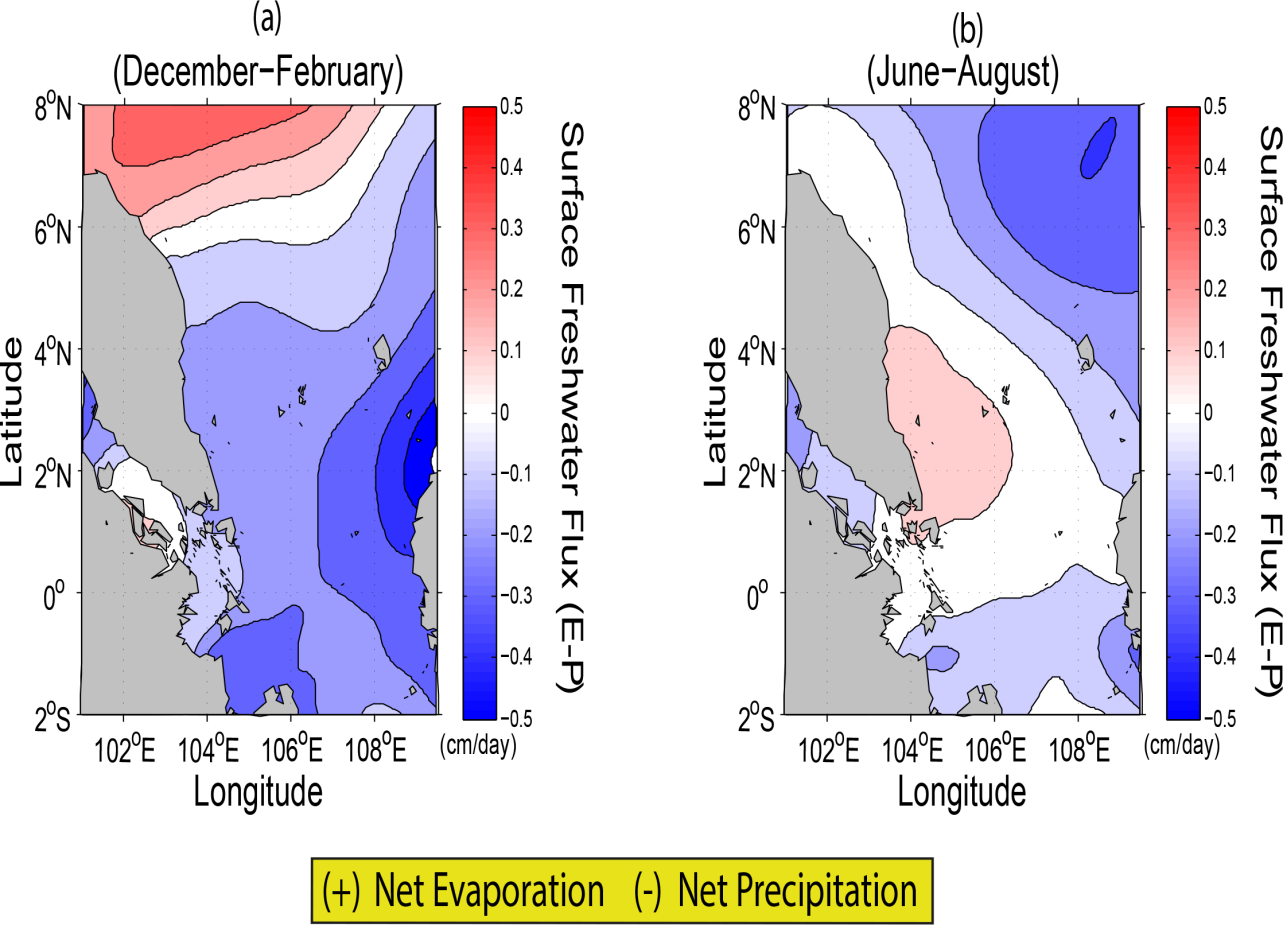
Seasonal variations in surface freshwater flux (cm/day) computed from monthly climatological data [23] based on amounts of Evaporation (E) and Precipitation (P) for (*a*) winter and (*b*) summer.

Seasonal variations of sea surface salinity (SSS) in the SSCS range between 32 and 34 psu [27]. The changes of SSS are due mainly to the effects of Evaporation (E), Precipitation (P), advection, convection, and mixing. The significant role of the seasonal variations of surface freshwater flux (E-P) in controlling the seasonal variations of SSS in the region is clearly shown in Fig 8. It is interesting to note that during winter (December-February) and summer (June-August), both the simulation and observation indicate the relatively low values of SSS averaging approximately 31 psu in the SM (not shown). This is likely due to the surface freshwater flux (E-P) input in the corresponding area in which net precipitation is high (Fig 8). Moreover, gradual change in salinity can be due to mixing between waters of different salinities, particularly in the summer season along the southern coastal areas of the ECPM.

Appropriate numerical study is needed to identify the individual effects of surface freshwater flux (E-P), wind stress and heat flux (such as solar radiation) in the SSCS. Hence, four numerical experiments as shown in Table 3 are conducted to isolate individual effects of each of the forces. These experiments include one control run (Run1) and three experiment runs with the same configurations and integration period of 10 years inclusive of 3 years spin-up but different sea surface forces. For Run1, comprehensive physical processes, including wind stress, surface freshwater flux (E-P), heat fluxes and open boundary forces are taken into account. For Run2, the surface freshwater flux is excluded from the control run. In the respective Run3 and Run4, wind stress and solar radiation as surface forces are not considered.

**Table 3:** Numerical experiment schemes performed in the present study.

Experiments	Wind stress	Surface freshwater flux (E-P)	Solar radiation flux
Run 1	Yes^a^	Yes	Yes
Run 2	Yes	No^b^	Yes
Run 3	No	Yes	Yes
Run 4	Yes	Yes	No

^a^ the effect is considered in the simulation

^b^ the effect is excluded in the simulation.

**Fig 9:**
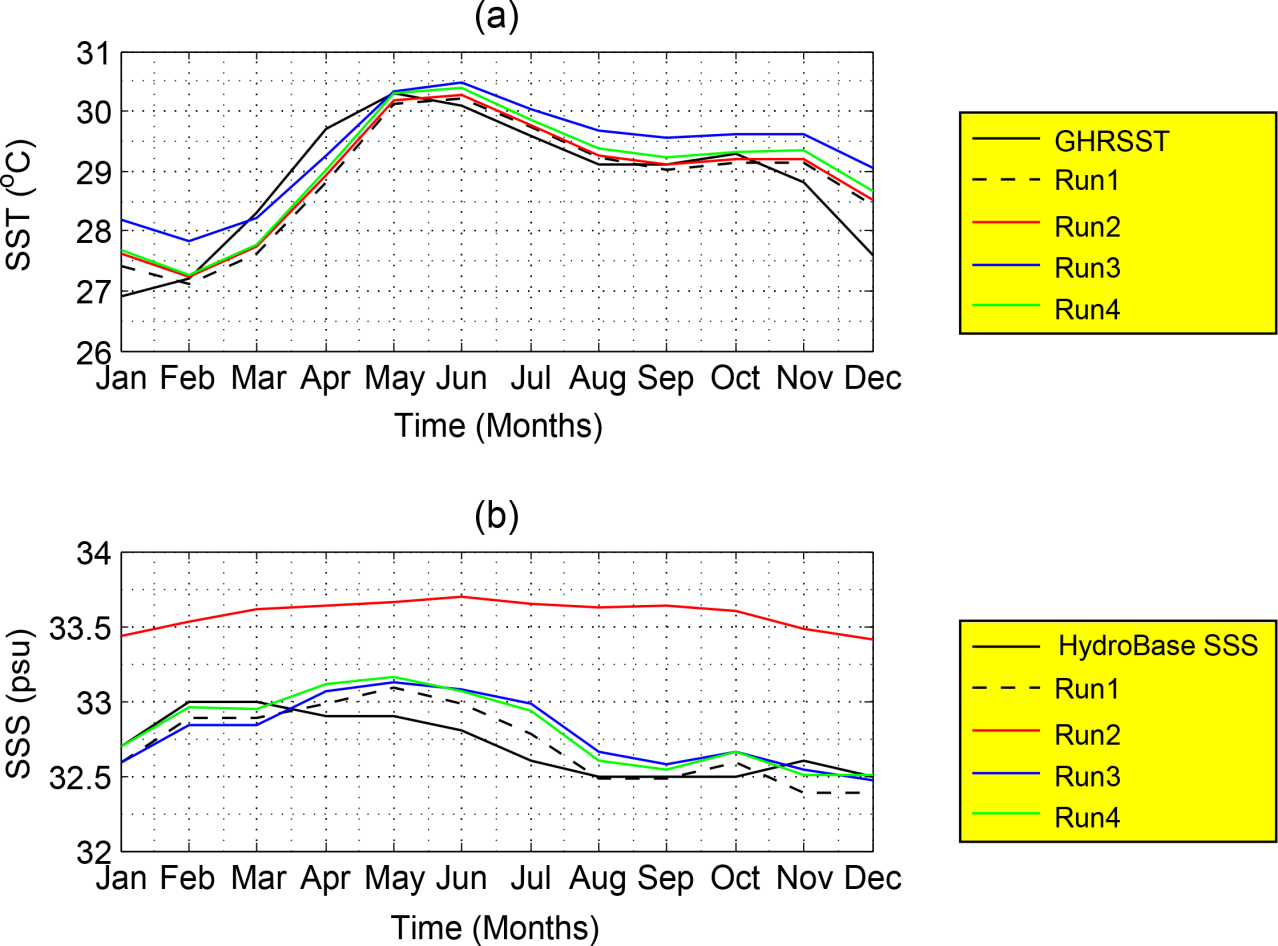
The mean monthly variations of SST (°C) and SSS (psu) represented for the GHRSST and HydroBase SSS (the black lines), control run (Run1, black dashed-line), surface freshwater effect run (Run2, red line), wind stress effect run (Run3, blue line) and solar radiation effect run (Run4, green line).

The mean monthly variations of SST and SSS derived from the observations of GHRSST, HydroBase SSS, respectively, and those from Run1 and Run2 to Run4, for averaged area between 101°E -109.5°E and 2°S-8°N are shown in Fig 9. The variations of both SST and SSS from the control run appear to be similar to those obtained from GHRSST and HydroBase SSS respectively. The sea surface temperature attains a minimum of 27°C (in January during winter) and a maximum of 30.5°C (in June during summer) (Fig 9A). The maximum peak of SSS occurs prior to April with the value of approximately 33 psu. It gradually decreases from the months of July to September to the lowest value of 32.5 psu (Fig 9B). The minimum SST is found to be in consonance with the maximum SSS (Fig 9) in February during winter. This could be due to the dominant effect of peak winter monsoon period on the variations of SST and SSS in the SSCS. However, comparison of variations between SST and SSS derived from observations and the control run with those from the experiment runs clearly show the strong effect of surface freshwater flux (E-P) in regulating the salinity changes in the study area. This implies that the lack of surface freshwater flux would cause not only the significant shift in the variation of salinity but also the changes in density gradient, signifying its important impact in altering the thermohaline circulations in the SSCS.

## Seasonal and Mean Annual Transports

In this section, we discuss the seasonal and the mean annual transports in terms of volume, freshwater, heat, and salt exchanges between the SSCS and the surrounding seas through the SS and the SM. These transports affect the mechanism and the formation of thermohaline circulation in the upper layers. To facilitate the computation of these transports, we specify the transect SS as the line joining A1 and A2 between the southern end of the Peninsular Malaysia and western Borneo whereas the transect SM is the line joining B1 and B2 located in the southern fringe of the SM as shown in Fig 1B.

### Seasonal transports

The volume transports are calculated from the sea surface to the seabed in the Cartesian coordinate system by the following equation,

$$VT=\int_{-h}^{0}\int_{l_{1}}^{l_{2}}\vec{v}.dl\;dh$$ (3)

where *VT* denotes the volume transport (10^6^ m^3^/s) and$\vec{v}$ (m/s) is the velocity vector. *h* (m) represents the total depth, *l*_*1*_ and *l*_*2*_ in the units of meter, are the respective lengths of the transects in the SS (i.e., A1A2) and the SM (i.e., B1B2).

**Fig 10:**
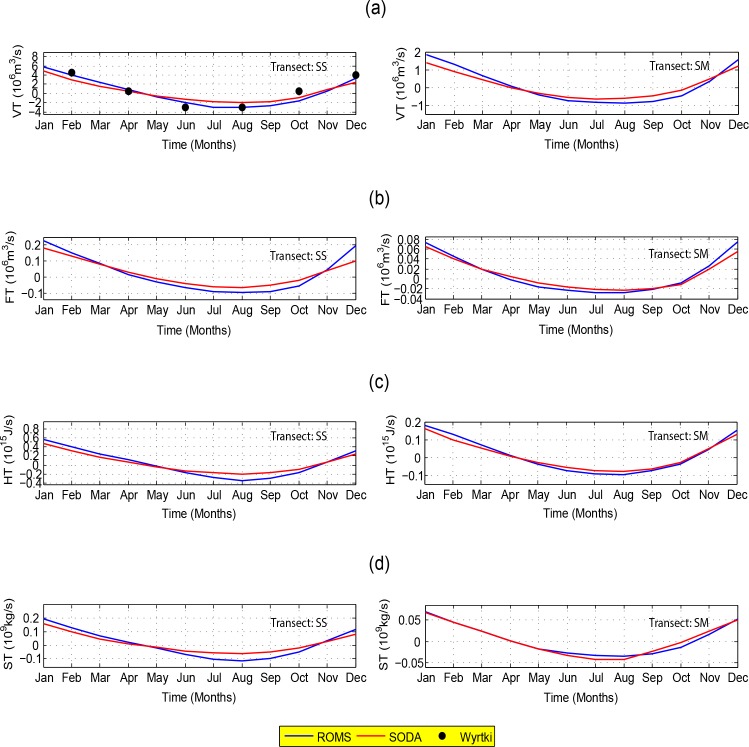
Seasonal transport of (*a*) volume, (*b*) freshwater, (*c*) heat, and (*d*) salt through transects in the SS and the SM based on ROMS (the blue line) and SODA (the red line). The circles in (*a*) indicate the bi-monthly observed volume transport by Wyrtki, [6]. Positive and negative values indicate outflow and inflow, respectively.

The seasonal transport of volume derived from ROMS is in good agreement with those of SODA and the bi-monthly observed data from Wyrtki, [6] which is only available for the SS (Fig 10A). It clearly reveals that inflow begins in April, reaches maximum (~3×10^6^ m^3^/s for the SS and ~1×10^6^ m^3^/s for the SM) around July, changes to outflow in October and reaches its maximum (~6×10^6^ m^3^/s for the SS and ~2×10^6^ m^3^/s for the SM) in January. This reflects the dominant role of monsoonal winds in causing volume outflow during winter and volumn inflow during summer.

The freshwater transport for transects SS and SM is given by the following integration:

$$FT=\int_{-h}^{0}\int_{l_{1}}^{l_{2}}(\frac{S_{0}-S}{S_{0}})\vec{v}.dl\;dh$$ (4)

where *S* (psu) is salinity for a particular water mass and *S*_*0*_ is a reference value set to 34.544 [10]. As shown in Fig 10B, the pattern of the seasonal freshwater transport derived from the ROMS and SODA through transects SS and SM generally follows that of the volume transport. Similarly, inflow begins in April, reaches maximum (~0.1×10^6^ m^3^/s for SS and ~0.03×10^6^ m^3^/s for SM) around August, and changes to outflow in October with its peak (~0.2×10^6^ m^3^/s for SS and ~0.07×10^6^ m^3^/s for SM) in January. The computed maximum freshwater outflow of 0.2×10^6^ m^3^/s for SS is comparable with the observed value which was measured by Fang *et al*. [7] for a very specific period in boreal winter of 2007–2008 using the Acoustic Doppler current profiler.

In terms of heat transport, the following equation is used:

$$HT=\rho_{0}C_{p}\int_{-h}^{0}\int_{l_{1}}^{l_{2}}(T-T_{0})\vec{v}.dl\;dh$$ (5)

where *ρ*_*0*_ ~1023 kg/m^3^ is the water density, *C*_*p*_ the specific heat estimated for particular water mass based on calculations by Millero *et al*. [35], *T* denotes the seawater temperature and *T*_0_, the reference temperature set to 3.72°C [7, 36]. In general, the estimated seasonal transport of heat through transects SS and SM for the ROMS and SODA are consistent with each other (Fig 10C). The estimated maximum heat outflow through transect SS in January is approximately 0.45×10^15^ J/s which is in accord with the observed values of Fang *et al*. [7]. For the transect through the SM, the maximum outflow towards the Andaman Sea is roughly 0.2×10^15^ J/s and the maximum heat inflow of about 0.1×10^15^ J/s (Fig 10C) occurs around August.

The salt transport is given by the following equation;

$$ST=\rho_{0}\int_{-h}^{0}\int_{l_{1}}^{l_{2}}S\vec{v}.dl\;dh$$ (6)

The estimated maximum outflows of salt through the transects SS and SM both occur in January. It has an approximate rate of 0.2×10^9^ kg/s (comparable to the observed value of Fang *et al*. [7]) through the SS and 0.06×10^9^ kg/s through the SM. Similarly, the maximum inflows of salt through the SS and SM occur in August with the respective rates of 0.1×10^9^ kg/s and 0.04×10^9^ kg/s.

In general, the rates of all the above transports in winter are greater than those in summer. In winter, the flows originate from the deep basin into the shallow continental shelf. From the Bernoulli's principle, the above fixed volume of the water mass is flowing horizontally from the deep basin of high pressure to the Sunda Shelf of low pressure. Such pressure difference results in a net force on the volume of the water mass, thus accelerating it along the flows.

### Mean annual transports

The mean annual transports of volume, freshwater, heat, and salt through transects SS and SM for ROMS and SODA are listed in Table 4. The differences between the estimated transports for SODA and ROMS may be due to the differences in horizontal and vertical resolutions, particularly in the shallow water region of SS and at the entrance of the SM [13]. As transports are a convection-diffusion-dominated process [37], model resolution obviously affects the process in terms of its sensitivity towards the turbulent flow in a coarser model. Perhaps, as suggested by *Ye and McCorquodale* [37] a more complex stress scheme, such as the Reynolds stress scheme [38] can be used to obtain directly the unknown turbulent stress to overcome the overestimation problem.

**Table 4:** Mean annual transports of volume (*VT*), heat (*HT*), freshwater (*FT*), and salt (*ST*), through transect SS and in the braket for transect SM. Positive values indicate outflow transports.

Reference	*VT* (10^6^ m^3^/s)	*HT* (10^15^ j/s)	*FT* (10^6^ m^3^/s)	*ST* (10^9^ kg/s)
ROMS	+0.32[+0.14]	+0.032[+0.014]	+0.023[+0.009]	+0.010[+0.0043]
SODA	+0.42 [+0.13]	+0.042[+0.013]	+0.026[+0.009]	+0.016[+0.0041]

As shown in Table 4, for both the ROMS and SODA, the transports throughout the year through transect SM are as equally significant as those through the SS. The estimated mean annual transport of volume and heat through transect SM into the Andaman Sea is about 44% of outflow through transect SS while those of freshwater and salt are about 39% and 43% respectively.

## Summary and Conclusion

We use a three dimensional regional ocean modeling system forced with the relevant oceanic and atmospheric variables to simulate the dynamics of the water circulations and to estimate the seasonal and annual variations of volume, freshwater, heat and salt transports through the corresponding transects in the SSCS and the Strait of Malacca. The characteristics of the simulated ocean dynamics in the region are found to be in agreement with the observed values (such as OSCAR, AVISO, GHRSST, HydroBase) and SODA. This reveals that the ROMS model is well calibrated for use in the SSCS. The water circulations during winter and summer are shown to be cyclonic and anticyclonic respectively with their associated eddies of different horizontal scales. The strong current along the ECPM during summer is noted to veer towards the east in the form of anticyclonic circulation due to the conservation of potential vorticity. Furthermore, the water circulation patterns in the SSCS north of 5°N are dominated by both of the geostrophic currents and the pressure gradient force. Those patterns south of 5°N are due solely to the wind stress in the presence of negligible Coriolis force. The seasonal exchanges of various transports through the main passages in the SSCS follow the patterns of water circulations. The seasonal transports through the SS are greater as compared to those of the SM due to the relatively larger width of the passage in the SS for the exchange to flow between the SSCS and the Java Sea. However, the mean annual transports show outflow occurs through the main passages in the SSCS into both the Java Sea and Andaman Sea. In addition, estimates of mean annual transports indicate the comparable importance of both the passages in the SS and SM. In comparison with the values of the transect SS, the percentages of estimated mean annual transports through transect SM range from 39% to 44%, emphasizing equally the importance of the Strait of Malacca as an inter-ocean transport passage in the SSCS. To date, the mechanisms involved in the distribution of primary productivity and net uptake of gases such as O_2_ and CO_2_ in the SSCS are still not clearly understood. This study can thus fill the lacuna to assist in assessing the changes, e.g., the stated net uptake of O_2_, CO_2_, which can influence the distribution of the nutrient balance in regulating the changes in the marine ecosystem.
